# The Protein Phosphatase 7 Regulates Phytochrome Signaling in *Arabidopsis*

**DOI:** 10.1371/journal.pone.0002699

**Published:** 2008-07-16

**Authors:** Thierry Genoud, Marcela Treviño Santa Cruz, Tea Kulisic, Francesca Sparla, Christian Fankhauser, Jean-Pierre Métraux

**Affiliations:** 1 Center of Integrative Genomics, University of Lausanne, Lausanne, Switzerland; 2 Department of Biological Sciences, Florida Gulf Coast University, Fort Myers, Florida, United States of America; 3 Department of Biochemistry, Faculty of Chemical Technology, University of Split, Split, Croatia; 4 Laboratory of Plant Physiology, Department of Biology, University of Bologna, Bologna, Italy; 5 Department of Biology, Unit Plant Biology, University of Fribourg, Fribourg, Switzerland; Massachusetts General Hospital, United States of America

## Abstract

The *psi2* mutant of *Arabidopsis* displays amplification of the responses controlled by the red/far red light photoreceptors phytochrome A (phyA) and phytochrome B (phyB) but no apparent defect in blue light perception. We found that loss-of-function alleles of the protein phosphatase 7 (AtPP7) are responsible for the light hypersensitivity in *psi2* demonstrating that AtPP7 controls the levels of phytochrome signaling. Plants expressing reduced levels of AtPP7 mRNA display reduced blue-light induced cryptochrome signaling but no noticeable deficiency in phytochrome signaling. Our genetic analysis suggests that phytochrome signaling is enhanced in the AtPP7 loss of function alleles, including in blue light, which masks the reduced cryptochrome signaling. AtPP7 has been found to interact both in yeast and *in planta* assays with nucleotide-diphosphate kinase 2 (NDPK2), a positive regulator of phytochrome signals. Analysis of *ndpk2-psi2* double mutants suggests that NDPK2 plays a critical role in the AtPP7 regulation of the phytochrome pathway and identifies NDPK2 as an upstream element involved in the modulation of the salicylic acid (SA)-dependent defense pathway by light. Thus, cryptochrome- and phytochrome-specific light signals synchronously control their relative contribution to the regulation of plant development. Interestingly, PP7 and NDPK are also components of animal light signaling systems.

## Introduction

Signal regulation is essential in perceptive systems. The control of continuous or intermittent signals (*i.e.* signal tuning, termination, maintenance, and oscillation) plays a central role in the organization and survival of a cell. Photoperception in *Arabidopsis* represents a challenging field for the investigation and understanding of the basic molecular mechanisms involved in signal processing.

The spectral composition, duration/period, and intensity of light have a direct impact on the fitness of plants. Consequently they have evolved towards the optimization of photon capture, adapting their morphology, development, and metabolism to the light conditions [1]. This is achieved through the continuous integration of information corresponding to biotic and abiotic parameters, and through metabolic adjustments specific to each particular phase of the life cycle [2]. It requires the precise interpretation and tuning of related signals by a molecular apparatus committed to cell information processing.

The specificity of the light receptors for a precise wavelength is not perfectly delimited in plants, yet in *Arabidopsis* one can distinguish a red light receptor called phytochrome B (phyB) from a related receptor called phytochrome A (phyA) by the specific activation of the latter through for instance exposure to far-red light only [1], [3]. These two photoreceptors contain the same tetrapyrrole chromophore, act as reversible switches in dimeric association, and represent the major regulators of plant responses during deetiolation and during day-light exposure, as revealed by mutants lacking both phyA and phyB [4]. Three other phytochromes have been characterized in *Arabidopsis* [5], [6]. PhyD and phyE play a role similar to phyB [7], [8] but with a lesser importance. PhyC is semi-redundant with phyB but its activity has effects on both far-red and blue light perception [9], [10] mediating flowering and growth responses [11]. Plants contain additional photoreceptors: phototropins phot1 and phot2 are the blue light receptors controlling phototropism, chloroplast movement and stomatal aperture, whereas cryptochrome (cry) receptors respond to high fluences of blue light during deetiolation (cry1), or to low blue light fluences during deetiolation with an involvement in the photoperiodic induction of flowering (cry2) [5], [12], [13]. The functions regulated by cry receptors are often similar to the processes controlled by phytochromes; however, several responses are modulated differently by the two classes of photoreceptors, which can act synergistically, specifically or antagonistically [13].

A recent description of the architecture of the phytochrome signaling network delineates three main signal routes regulated by positive and negative factors [14], [15], [16]. Whereas phyA and phyB each activate a specific sub network, a common pathway non-specifically induced by both phytochromes also contributes to the regulation of the light-controlled genetic network. Several proteins have been shown to intervene in more than one receptor signaling pathway. For instance SUB1 [17] and the zinc finger protein HRB1 [18] negatively regulate cryptochrome and phytochrome signaling, while the transcription factor OBP3 is a positive regulator of phyB and negative regulator of cry1 signaling pathways [19].

Cross-talks also take place between light perception and other cellular functions; for example, phytochrome signals modify the expression of auxin-regulated genes and proteins [20], [2] and control the expression of gibberellin-related genes by modulating the level of the phytochrome-interacting bHLH factor PIL5 [21]. The phyA and phyB signaling pathways also activate the pathogen defense responses controlled by SA in *Arabidopsis* [22]. Moreover, phytochromes modulate the expression of several cold-related genes through the C-repeat/dehydration responsive element (C/DRE) [23]. The activity of phyB is fine-tuned by the cytokinin response regulator ARR4 in a reciprocal mode of regulation [24] and a transcription factor (MYC2) acts as a common element of convergence of the abscisic acid, jasmonic acid, and light signaling networks [25].

We have previously isolated a light hypersensitive mutant (*psi2*) defining a negative regulator of a common signaling pathway controlled non-specifically by phyA and phyB [26]. The *psi2* mutant contains wild type levels of these phytochromes and shows an apparently unaltered sensitivity to blue light [26]. We have now isolated and identified the wild type gene corresponding to *psi2* and studied how the related protein regulates the levels of phyA and phyB signals.

## Results

### Identification of PSI2 as AtPP7

**Figure 1:**
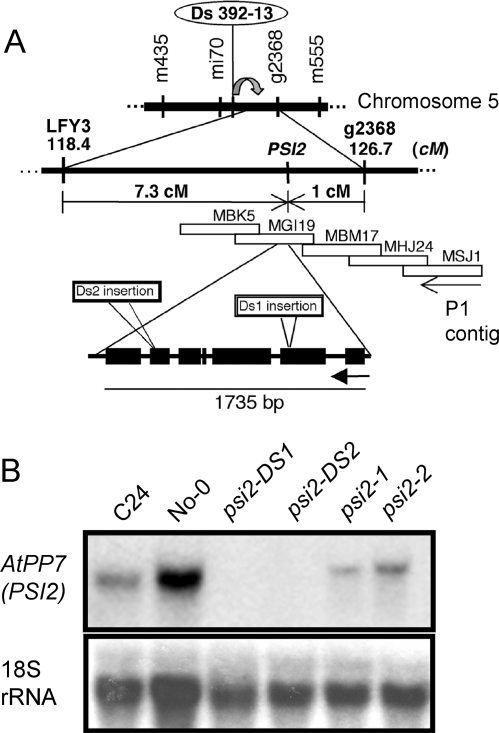
Chromosome 5 contains the *psi2* locus and the gene responsible for the *psi2* phenotype. (A) Rough mapping has located the *psi2* mutation between markers LFY3 and g2368 in the C24 ecotype. Transposition of a mapped Ds-element present in the line Ds-392-13 (No-0 ecotype) was induced to identify the *PSI2/AtPP7* gene. The Ds sequences in *psi2-DS1* and *psi2-DS2* plants allelic to *psi2-1* has reinserted in the region corresponding to P1 clone MGI19. (B) RNA gel blot analysis of total RNA extracted from WT and *psi2* mutants. The expression of the *MGI19.6* gene corresponding to the *AtPP7* gene was absent in the DS1 and DS2-transposon-induced *psi2* mutant. Each line contained 20 µg RNA.

Coarse mapping had located the *psi2* locus between two markers at the bottom of chromosome 5 [26]. Using ecotype polymorphism detection, the site of the *psi2* mutation was narrowed to a region shorter than 1 cM (Figure 1A). A strategy of inducible transposition was selected to identify the *PSI2* gene. *Arabidopsis* lines containing a single inducible transposon system (Ds 392-13, located at 9.4 cM upstream of the *psi2* locus) were crossed with transposase-containing plants to generate random selectable transpositions [27]. Plants positive for transposition, and presenting visible characteristics similar to the EMS *psi2* mutant phenotype were isolated. Two candidates combining both characters were identified (*psi2-DS1*, *psi2-DS2*). These lines were then backcrossed in WT and tested for allelism with the EMS *psi2* mutants. Examination of the F1 and F2 generations revealed that both transposon-insertion mutants were recessive and allelic to the EMS mutants for all the phenotypic characteristics previously identified. PCR amplification with specific nested primers [28], [29] of the circularized DNA adjacent to the Ds transposon insertion site in *psi2-DS1* identified a fragment of sequence corresponding to a leftward transcription unit called *MGI19.6*, and annotated as the Serine/Threonine protein phosphatase (Kazusa DNA Research Institute, Chiba, Japan) named AtPP7 [30]. The Ds insertion in the second allelic transposon mutant *psi2-DS2* was located near the right border of the sixth exon of the same gene (Figure 1A; data not shown). The level of *MGI19.6* messenger in the WT and mutant plants was determined with a cDNA probe (from EST 122A12T7) through RNA gel blot analysis; results confirmed that both *psi2-DS1* and *psi2-DS2* are complete knock-out mutants (Figure 1B). The two EMS alleles *psi2-*1 and *psi2-2* display less transcript than the isogenic related C24 ecotype, suggesting that the loss of AtPP7 activity in the chemically-induced alleles may be due to protein sequence variation. This was confirmed by sequencing of the At5G63870 locus in those alleles. In *psi2-1* a G to T substitution led to an arginine to lysine substitution, whereas in *psi2-2* the addition of a G caused a frameshift in the sequence.

**Figure 2:**
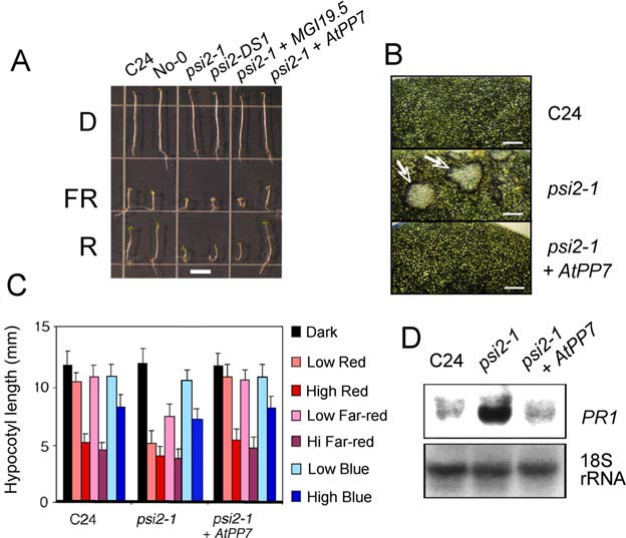
Complementation of the *psi2* mutation by a genomic sequence of *MGI19.6*/*AtPP7* gene . (A) Morphology of WT seedlings, *psi2* mutants, a transformed *psi2* mutant plant containing a genomic sequence of *AtPP7*, and a *psi2* mutant transformed with the control gene *MGI19.5*. Plants were grown for 5 days in continous light. Fluence rates in µmol m^−2^ s^−1^: far-red (FR), 5; red (R), 8; D, darkness. Bar: 5 mm. (B) The red light induction of HR-like lesions (arrows) on leaves of *psi2* were abolished by the presence of an intact genomic sequence of *AtPP7*. Plants were exposed to 50 µmol m^−2^ s^−1^ continuous red light at constant 20°C temperature. Bar: 0.7 mm. (C) The hypersensitivity to red and far-red light-induced block of hypocotyl elongation is suppressed in *psi2-DS1* transformed with intact *AtPP7*. Seedlings were grown 5 days under the following light exposure (fluence rates in µmol m^−2^ s^−1^: low red, 1; high red, 40; low far-red, 0.4; high far-red, 13; low blue 2; high blue, 38. (D) Amplification of SA-dependent *PR1* induction in the mutant *psi2* is complemented by an intact genomic sequence of *AtPP7*. Plants were grown for 2 weeks under 10 µmol m^−2^ s^−1^ white light at 22°C and then transferred to continuous red light of 30 µmol m^−2^ s^−1^ during 48 h at the same temperature.

The identification of *PSI2* as the *AtPP7* gene was confirmed by complementation of *psi2-1* and *psi2-DS1* with a 2.8-kb genomic fragment corresponding to the *MGI19.6* gene (AT5G63870). The control transformations consisted of both an empty vector and the most proximal gene called *MGI19.5* (AT5G63860) annotated as a GTP/GDP exchange factor. Results showed that only lines containing *MGI19.6* (genomic *AtPP7*) completely revert to WT the *psi2* phenotype (Figure 2; Figure S1). Reversion of the hypersensitivity to red and far-red light was further examined in the complemented *psi2* plants (Figure 2, Table S1, and Figure S3). These plants were first subjected to high fluence of red light and examined for the presence of light-inducible and phytochrome-dependent HR (Hypersensitive Response) on leaves using microscopy (Figure 2B). Both HR induction and *PR1* expression induced by light were reverted in the *MGI19.6*-complemented *psi2* lines (Figure 2B, 2D). The hypocotyl elongation responses under different wavelengths of light were measured for the complemented lines (Figure 2C), as well as the enlargement of cotyledons in red light (Figure S3). Since the complemented lines contain the *CAB2-luciferase* reporter gene, video imaging was used to monitor the expression of the luciferase gene for *CAB* gene induction after exposure to short red and far-red light pulses (Table S1) using the conditions described in [26]. In this phenotypic analysis, statistical complementation of the *psi2* phenotype was observed in lines containing *MGI19.6*. For instance, the *p*-value of the *t*-test comparing the elongation of C24 and the complemented *psi2-1* line (Figure 2C) is 0.63 in low far-red and 0.55 in low red light, indicating strong similarity. The sensitivity of the same lines to short light pulses was reduced to the WT level (Table S1) further confirming that the presence of intact *MGI19.6* complements defects in the *psi2* mutants.

### AtPP7 interacts with nucleotide diphosphate kinase 2 (NDPK2)

**Figure 3:**
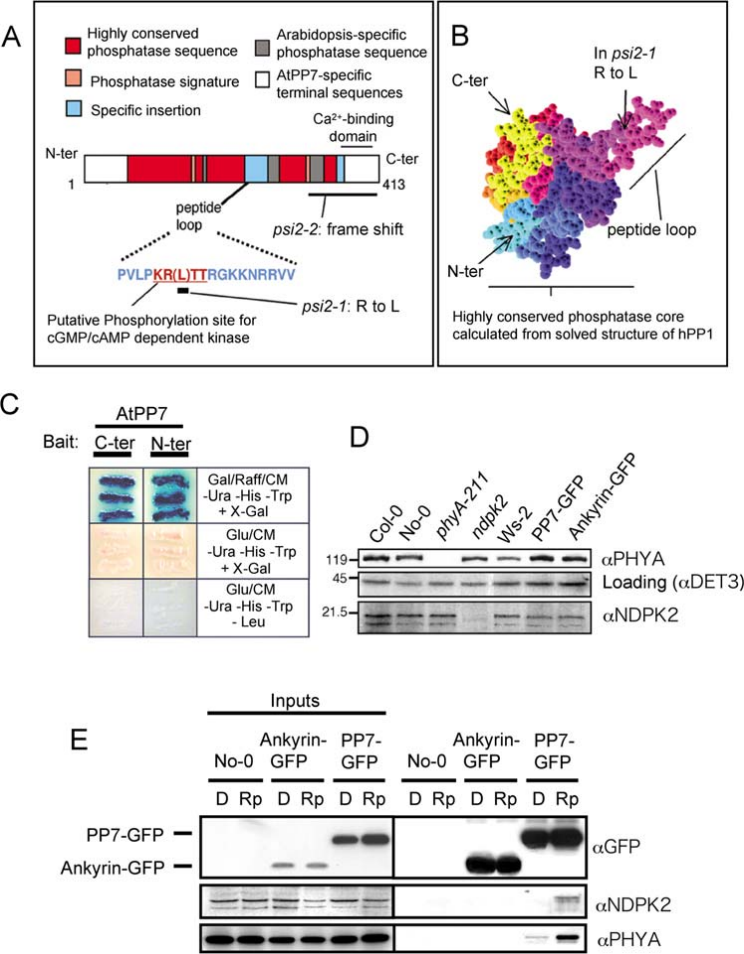
Structure of the AtPP7 protein and screen for AtPP7 interacting protein. (A) Location of EMS point mutations on the protein sequence and description of the conserved and non-conserved phosphatase domains. (B) Location of the peptide loop domain in the periphery of the main phosphatase core, as calculated from the highly similar domain of hPP1 using *Geno3D* [56]. (C) Yeast colonies positive in a yeast two-hybrid assay using the N- and C-terminus of AtPP7 as bait and an *Arabidopsis* cDNA library as preys. Both AtPP7 termini interact with a fragment of the *Arabidopsis* nucleotide diphosphate kinase 2 (NDPK2) protein. (D) Level of NDPK2 and phyA protein in *ndpk2* and *phyA* null mutant lines and their related wild types grown 4 days in darkness at 22°C. Western blot analysis were performed as described in text. (E) NDPK2 and phyA interacts with AtPP7 *in vivo*. Transgenic *Arabidopsis* expressing either a AtPP7-GFP fusion or a constitutive nuclear ankyrin-GFP fusion were grown 4 days in darkness and exposed to a 5 min pulse of red light 10 µmol m^−2^ s^−1^ at 22°C (D: dark control; Rp: red pulse). Protein extracts from these plants were incubated with agarose-bound anti-GFP antibodies. Co-immunoprecipitated NDPK2, phyA and DET3 were then detected after SDS-PAGE separation on nitrocellulose-blotted gel using specific antibodies. Input samples contained total protein extract.

To locate the site of action of the AtPP7 protein in the phytochrome signaling pathway, we conducted a screen for AtPP7-interacting proteins using a yeast two-hybrid system. Since the central part of the protein (Figure 3A) is highly similar to other phosphatases, only the N- and C-termini sequences of AtPP7 cDNA were used for a screen against a cDNA library of *Arabidopsis* [31]. Both identified a same interactor in the screen (Figure 3C), whose sequence encodes the *Arabidopsis* nucleotide diphosphate kinase 2 (NDPK2), a protein that has been shown to interact with phytochrome [32]. Since the interaction screen was conducted with only short portions of the AtPP7 protein, the interaction between the full length AtPP7 and the complete NDPK2 was further studied and confirmed using FLAG-tagged AtPP7 and 6xHis-tagged NDPK2 (Figure S4, Text S1) and tested *in vivo* using transgenic lines containing AtPP7-GFP protein fusion in the *psi2-DS1* background (Figure S2) and a coimmunoprecipitation strategy using anti-GFP antibody to precipitate proteins binding AtPP7-GFP *in vivo* in dark conditions and after a pulse of 5 min red light at 10 µmol m^−2^ s^−1^ (Figures 3D, 3E). An *Arabidopsis* line containing nuclear-expressed ankyrin-GFP fusion has been used as negative control in this experiment. Results of these assays confirm that NDPK2 binds AtPP7 *in vivo* in a light dependent manner, since no binding is detected in darkness, whereas a pulse of red light induces the association of the two proteins (Figure 3E). Coprecipitation of phyA in the same assay suggests that AtPP7-GFP binds to NDPK2 associated with the light-activated form of phyA, most likely after transport of the complex in the nucleus, as indicated by the strong reduction of this association in darkness, when phyA is mostly cytoplasmic [33].

### *ndpk2* is epistatic to *psi2* in the regulation of phytochrome signaling

**Figure 4:**
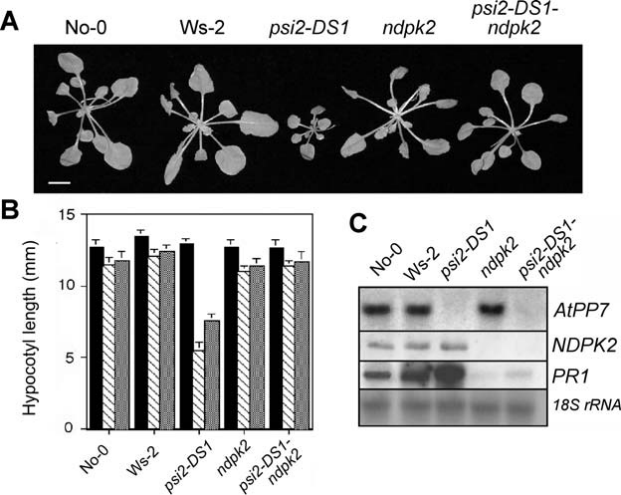
AtPP7 controls the level of phytochrome signals through NDPK2. (A) Phenotype of *psi2-DS1* (in No-0 ecotype) and *ndpk2* (in Ws-2 ecotype) versus the double mutants *psi2-DS1-ndpk2* under continuous white light exposure (20 µmol m^−2^ s^−1^). (B) Reversion of *psi2* phenotype in the *ndpk2* mutant background. Plants were exposed to continuous low fluence rate of red light (1 µmol m^−2^ s^−1^, shaded boxes) and far-red light (0.4 µmol m^−2^ s^−1^, grey boxes) for 7 days before measurement of hypocotyl elongation. Dark control: black boxes. (C) NDPK2 controls the modulation of SA-induced *PR1* gene expression by light. 3 week-old plants of the described genotypes have been treated with 250 µM SA; total RNA was extracted 48 h after treatment (80 µmol m^−2^ s^−1^ white light) and used for RNA gel blot analysis. Each line contains 20 µg RNA.

A double mutant *psi2-DS1 ndpk2* was produced to determine whether the modulation of phytochrome signals by AtPP7 depends on its interaction with NDPK2. In this double mutant, a clear reversion of the phenotypic reduction of cell elongation in the *psi2-DS1* mutant was observed in continuous white light (Figure 4A), as well as in continuous low red light (Figure 4B), a discriminating condition for the involvement of both phyA and phyB in the *psi2* phenotype [26]. This reversion notably affected both the expression of the *PR1* gene after treatment with 250 µM SA (Figure 4C) and the formation of HR at high light (data not shown). Besides its activity in the regulation of development by phytochrome, NDPK2 is also likely to be the upstream component of the phytochrome pathway controlling the switching and modulation of the SA-dependent defense pathway by light [22].

### Increased activity of phytochrome signaling in *psi2* alleles of AtPP7

**Figure 5:**
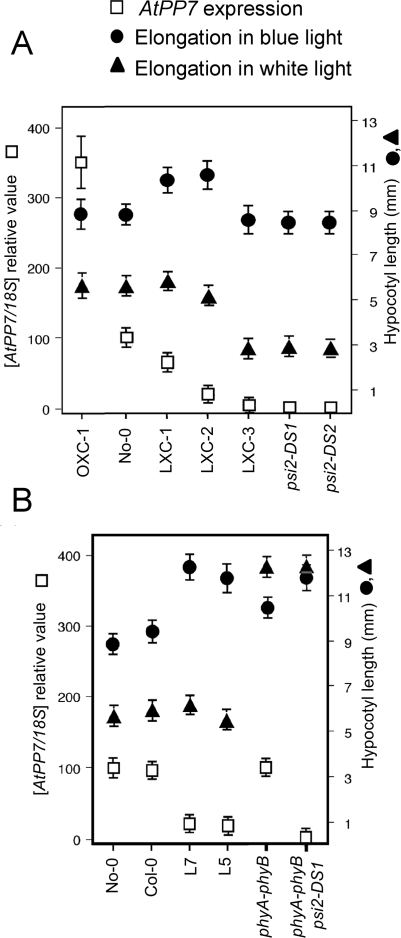
Blue-light perception defect at decreasing levels of *AtPP7* expression is compensated under a triggering threshold. (A) Different lines of *psi2-DS1* mutants transformed with a 2.8-kb genomic fragment of AtPP7 showing a lower *AtPP7* expression level than WT No-0 (LXC-1, LXC-2, LXC-3) or a higher level than No-0 (OXC-1) due to positional effect (open squares), have been exposed to continuous blue (10 µmol m^−2^ s^−1^; filled circles) or white light (40 µmol m^−2^ s^−1^; filled triangles) for 5 days before the measurement of hypocotyl length and *AtPP7* expression. (B) AtPP7 antisense lines L5 and L7 (in Col-0 ecotype) described in Møller *et al.* (2003), *phyA-phyB* and *phyA-phyB-psi2-DS1* multiple mutants were grown in the same light conditions as in panel (A). 30 µg of total RNA extracted from each line have been submitted to RNA gel blot analysis using cDNA fragment of AtPP7 as a probe, and quantitatively estimated by phosphorimaging. The values of *AtPP7* expression (open squares) have been normalized using 18S-rRNA amounts and expressed relatively to the No-0 content ( = 100). Note the reversion of the defect in hypocotyl elongation under blue light in complete absence of *AtPP7* in panel (A).

A former study has shown that the responses of *Arabidopsis* to blue light are dependent on the level of *AtPP7* expression [34]. Transgenic plants with reduced expression of *AtPP7* display a lower sensitivity to blue light, whereas overexpression of AtPP7 does not modify the perception of this light wavelength [34]. These results clearly assign to AtPP7 the role of a positive regulator of the blue light signaling pathway. In addition, AtPP7 also regulates the activity of phytochrome signaling pathway, since a complete absence of AtPP7 as in the *psi2-DS* mutants trigger a strong increase in the sensitivity of the plant to red and far-red light (Figure 2, Table S1), an effect which is not seen in antisense lines [34]. To test whether the different amount of AtPP7 in these plants caused distinct effects, we compared the blue light response of seedlings containing decreasing amount of AtPP7 expression (Figure 5). The *AtPP7* antisense lines (L5, L7), the lines of *psi2-DS1* mutants transformed with genomic sequence of *AtPP7* but expressing the transgene in lower amount than WT (LXC1, LXC2, LXC3), and the two *psi2-DS* mutants were exposed to 10 µmol m^−2^ s^−1^ of continuous blue light for 5 days and their response compared to their level of *AtPP7* expression (Figure 5A, Figure S5). Low expressors showed less sensitivity to blue light than WT No-0, whereas the plants with no detectable amount of AtPP7 presented a WT response for both hypocotyl elongation and anthocyanin accumulation. In order to determine whether phytochromes were involved in this recovery in blue light perception, we tested the response of a double mutant *phyA-phyB* and triple mutant *phyA-phyB-psi2-DS1* (Figure 5B). The *phyA-phyB* mutant displayed a reduced sensitivity to blue (*p*-value<0.001) and the triple mutant an even stronger insensitivity, comparable to the response of the AtPP7 antisense lines (the *p*-value of the *t*-test comparing the triple mutant elongation with the L5 and L7 line elongation in blue light is 0.80 and 0.12, respectively). In white light however, *phyA phyB psi2* had the same phenotype as *phyA phyB* (Figure 5) (*p*-value: 0.67).

## Discussion

Our analysis of *AtPP7* loss of function (*psi2-DS*) and hypomorphic alleles (antisense lines) reveals unexpected complexity in the function of this gene. *AtPP7* hypomorphic alleles display normal phytochrome signaling but reduced cryptochrome signaling [34]. Our data show that below a certain activity threshold *AtPP7* mutants present enhanced phytochrome signaling. Thus, we propose that the apparent absence of phenotype in *psi2-DS* alleles in blue light is due to the compensation of reduced cryptochrome signaling by increased phytochrome signaling. Indeed, phytochromes are known to be activated under several light wavelengths, notably blue light [35], [36]. Consistent with this hypothesis the *phyA-phyB-psi2-DS1* triple mutant has a stronger blue light phenotype than *phyA-phyB* double mutants.

The primary role of AtPP7 as a modulator of phytochrome signals is confirmed by several lines of evidence. Firstly, the activity of a common signaling pathway involving both phyA and phyB is increased in *psi2,* notably in etiolated seedlings and under light conditions that exclude a photosynthetic defect or the involvement of photo-oxidation [26], [22]. Secondly, the phytochrome signaling amplification and its related disorder in *psi2* are fully complemented by transformation with the WT *AtPP7* gene. Thirdly, AtPP7 is genetically epistatic to and interacts *in vitro* and *in vivo* with NDPK2, a positive element transducing light signals from phytochromes [32].

It is possible that AtPP7 modulates phytochrome signaling by dephosphorylating a specific isoform of the phytochrome molecule and/or a phosphorylated protein regulating phytochrome signals. Further investigation will be needed to identify the specific substrate of AtPP7. Interestingly, the light-regulated PP7-phytochrome interaction could be due to alternative phosphorylation states of phyA.

**Figure 6:**
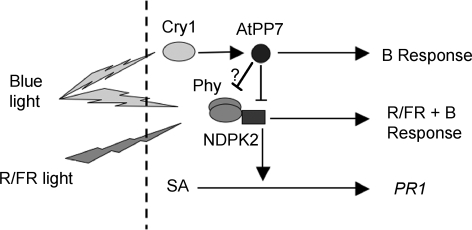
Model of the regulation of phytochrome signaling by AtPP7. Model of signaling connection involving AtPP7, as a positive regulator of cryptochrome signaling and negative regulator of phytochrome signaling through NDPK2. The interaction of phy and SA signaling for the control of PR1 expression is also represented. AtPP7 is thought to dephosphorylate one or several substrates, such as NDPK2, phytochromes or even cryptochromes, most likely in the nucleus.

In the case of the mutant *psi2-1*, a point mutation in the AtPP7 molecule is located within a putative kinase G phosphorylation sequence positioned inside a crucial regulatory region of the protein (Figure 3) [37], [38], and the mutation present in *psi2-2* produces a defect in the correct translation of the C-terminus region, a domain known to bind calmodulin or calcium directly [37], [38]. Therefore, it is reasonable to suggest that AtPP7 could be activated by both cyclic nucleotides and calcium. The analysis of the *sub1* mutant indicates an interaction between blue light perception through cry1 and phytochromes [17]. The calcium-binding protein SUB1 is a non-specific regulator of cry1 and phyA pathways. Hence, connection between the two classes of receptors may involve calcium signals. A simplified model of AtPP7 location in the light signaling pathway is proposed in Figure 6.

A consequence/function of the inter-pathway regulation is of particular relevance for the light-dependent SA-controlled defense signaling pathway. Our previous studies have shown that chloroplast activity is involved in the process of defense against pathogens in *Arabidopsis* [22]. Therefore, a block in blue light signaling could modify the control of photosynthetic activity or plastid development and consequently the response to pathogen aggression. This interpretation is supported by the recently described interaction of NDPK2 with the MAP-kinase system (MAPK3,6) [39], an important signaling element involved in the control of oxidative stress during the interaction of plants with a pathogen. Moreover, it has been shown [40] that a mutation in a defense response-related protein TIR-NBS-LRR, which has a dominant negative effect on phytochrome signaling, reduces defense ability against pathogenic *Pseudomonas*, further indicating that phytochrome signaling interacts with defense signaling.

AtPP7 is mainly located in the nucleus [37] (Figure S2), this is also the case for cry2 and cry1 in darkness, and for phytochromes under exposure to specific light conditions [13], [15]. NDPK2 has been shown to be nuclear and cytoplasmic in *Arabidopsis*, and to interact with proteins (phyA and phyB) that translocate to the nucleus under light exposure [32]. Therefore, interaction of the two proteins for the control of phytochrome signaling is likely to occur in the nucleus, in a light-dependent manner as suggested by the difference of binding between proteins present in the extract of dark-grown and light-treated seedlings (Figure 3E). An interaction of AtPP7 with NDPK2 associated to the Pfr form of phyA after nuclear transport of the photoreceptor is suggested by a stronger co-precipitation of phyA, AtPP7, and NDPK2 in red-light exposed samples. Interestingly, NDPK2 accumulates in mature chloroplasts of *Arabidopsis* protoplasts transformed with full-length or truncated NDPK2 gene constructs [41], whereas NDPK2 is not detected in the cytoplasm in this experiment due to technical limits [41]. The presence of NDPK2 in the nuclear compartment might hence be transient, phytochrome-dependent, and related to signaling activity during the dark to light transition. Further work will clarify this aspect.

The presence of cry receptors and of a PP7 in retinal cells [12], [42], [43], together with the regulation of the activity of a rhodopsin-interacting trimeric G-protein by NDPK [44] suggest that the nature and properties of the signaling components involved in light perception may have either converged among the different kingdoms or emerged from shared chloroplast-related ancestors as in Gehring's “Russian doll” model [45]. The defects in AtPP7 function in the *psi2* mutants strikingly produce a phenotype similar to light-triggered apoptosis observed in certain genetic diseases [46], and to the retinal degeneration diseases caused by an ill-timed activation of programmed cell death in different animals (Drosophila and mammals) [47], [48]. Recent work has shown that the number of light-triggered HR-like lesions on Arabidopsis plants lacking AtPP7 increased upon heat shock treatment (45°C, 30 min) [49], further suggesting that AtPP7 indirectly controls cell death mechanisms in plants. Further characterization of AtPP7 activity *in vivo* and *in vitro* will contribute to a better understanding of the biological mechanisms involved in the synchronization of coincident signals, and may bring new insights on the broad dynamics of signaling and its related disorders in plant and animals.

## Materials and Methods

### Plant growth conditions

Seeds of the various genotypes were routinely sterilized by soaking once in 70% ethanol for 1 min, and then in 10% hypochlorite solution containing 0.1% Tween 20 for 10 min. They were finally washed with three changes of sterile water, and sown on Murashige and Skoog (JRH Biosciences, Lenexa, KS) solid medium containing 3% sucrose. Plates were kept in the dark at 4°C for 4–5 days before germination. To synchronize seed germination, plates were exposed to 20–30 min white light (25 µmol m^−2^ s^−1^) after 3 days of imbibition in darkness. Fluorescent bulbs were used for continuous white light of various fluence rates (GRO-LUX F96T 12/GRO; Sylvania Co., Danvers, MA). The fluence rate was attenuated by covering the plates with a combination of paper filters. For direct growth on soil, seeds were sown on pasteurized soil (a 3∶1 mix of humus/perlite) wetted with distilled water, and the pots were subjected to the cold and dark conditions described above. Seeds were germinated and seedlings grown under a 16-hr light/8-hr dark cycle (at temperatures of 20°C during light exposure and of 16°C during the dark period), and/or in continuous light regime (bulbs F36 W/33, General Electrics Co., Cleveland, OH), red and far-red light were supplied by LED light sources (Quantum Devices, Barnfeld, WI). Far-red light was filtered through a Plexiglas layer (model 067894; West Lake Plastics, Lenni, PA). Continuous blue light was provided by F20T12-B fluorescent bulbs (General Electric Inc.) and filtered through two layers of Plexiglas (No. 2424; Dayton Plastics, Columbus, OH).

### Generation of transposition-induced mutants

To generate insertional psi2 mutants we used the Ac/Ds system [27] available at the ABRC stock center. A Ds transposon line (Ds5 392-13, CS8525; in No-0 ecotype), containing a unique T-DNA, created in the Nina Fedoroff's laboratory (Pennsylvania State University) and mapping between markers mi70 and g2368 at the bottom of chromosome 5, was crossed with a line containing an Ac-transposase (CS8533). Two thousand F1 seeds were grown in pools of fifteen, and three hundred F2 individuals obtained from these plants were selected for transposition and presence of a reinserted transposon following the method described at the Plant Transposon Laboratory web site (http/www.biotec.psu.edu/ptl). 7-day old plants surviving on the selective medium containing 20 g/l hygromycin and 6 ppm chlorsulfuron, were transferred to soil and grown under white light (100 µmol m^−2^ s^−1^) for phenotype characterization. Plants displaying short hypocotyls and light-dependent HR (hypersensitive response)-lesions under these conditions were isolated and grown on soil separately for tests of allelism and dominance. Subsequently, 50 seeds of each of the selected F3 plants were tested on Murashige and Skoog medium containing naphthalene acetamide (Sigma) to determine the presence of Ac-transposase.

### Inverse PCR

Recessive transposition-induced mutants allelic to EMS-generated *psi2* plants and containing no Ac-transposase were grown under low light fluences. DNA was extracted from these plants and digest with restriction enzymes *Sau*3A, *Cfo*I, *Bst*YI, *BstY*I+*Bcl*I, as described [29]. Digested fragments were ligated to form circular fragments and a DNA sequence corresponding to the adjacent border of the Ds-transposon was amplified by inverse PCR using the primers B38: GATATACCGGTAACGAAAACGAACG and B39: TTCGTTTCCGTCCCGCAAGTTAAATA, followed by a subsequent amplification with the nested primers D71: CGTTACCGACCGTTTTCATCCCTA and D75: ACGAACGGGATAAATACGGTAATC. The positive fragment obtained from *Cfo*I digestion, was cloned into pCR2.1 for amplification and sequencing.

### Complementation analysis

A 2789-bp portion of the genomic sequences of *AtPP7* (AT5G63870), including the promoter region, was PCR-amplified using Takara LA Taq polymerase (Takara Shuzo Co., LTD., Shiga, Japan) and primers R: CCCCATCTTTTCTTCACTTTCGAA and F: CCTGACAAAACAGAGCAAAATCTTACA. The amplified product was cut with *Eco*RI and *Xba*I, and the resulting 2577-bp fragment was inserted into pGEM-T Easy (Promega Biosciences, Inc., San Luis Obispo, CA, USA) and amplified in *E. coli*. The genomic sequence was then excised from the recombinant plasmid with *Eco*RI, gel purified and cloned into a dephosphorylated binary plasmid (pBINPLUS), and sequenced for confirmation of integrity. This recombinant plasmid was subsequently introduced into *Agrobacterium* by electroporation and the transformed bacteria were used to transfect low-light grown *psi2-1* and *psi2-DS* mutant plants by floral dipping with modifications, as described [28]. A genomic sequence of the AtPP7 closest gene on the chromosome, with its promoter region (AT5G63860) was amplified by PCR with primers F: TTGAATTCAAACATAGCGGT and R: TTTCCATATGAGAACCATGCAT, cloned into pCR2.1 (Invitrogen Corp., CA, USA) and transferred into the binary plasmid. The recombinant plasmid as well as an empty binary plasmid vector were introduced in parallel into *psi2* plants. Seedlings positive for transformation were transferred to soil; the resulting plants were selfed, and also crossed with EMS-generated *psi2* plants. M2 individuals of the transformed lines expressing AtPP7 at a WT level were grown under 100 µmol m^−2^ s^−1^ white light and their leaves screened under the microscope for detection of light-inducible lesions. Hypocotyl elongation of 7-day old M2 plants from the transformed lines was measured after a continuous red light treatment (30 µmol m^−2^ s^−1^). The luciferase gene controlled by the *CAB2* promoter was introduced into the *psi2-1* line complemented with genomic *AtPP7* by crossing this line with a *psi2-1* plant containing the reporter transgene see in [26]; F3 lines with a WT morphology were used for light pulse experiments. Seedlings grown in darkness (150 per essay), were briefly illuminated with a pulse of light of the different wavelength and returned to darkness. The light emission of luciferin-treated plants was scored 20 h after the pulse by video imaging and computer image analysis as described [26]. Homozygous lines of *psi2-DS1* expressing the transgene at a lower amount than the No-0 WT (lines LXC-1, 2, 3) or at a higher amount (OXC-1) due to positional effect were selected for hypocotyl length and anthocyanin measurement in various light conditions.

### RNA gel blot analysis

We performed RNA extraction and RNA gel blot analysis as described [22]. *PR1* probe was a *Xho*I-*EcoR*I fragment of the coding sequence cloned into pBluescript (Stratagene); the vector containing *PR1* probe was a gift from the laboratory of John Ryals (Metabolon, Inc., Research Triangle Park, NC). As loading control for total RNA, we used an 18S rDNA fragment cloned from *Arabidopsis* (using primers F: TCTGATGATTCATGATAACTCGACG and R: GCAAGGTGTGAACTCGTTGAAT). Hybridization were quantified using a Phospho-Imager (BIO-RAD GS-525) coupled to Molecular Analyst software (Biorad Laboratories, Inc.).

### Hypocotyl length and cotyledon surface measurements

Hypocotyl lengths were measured on projected pictures of seedlings, which were transferred and placed horizontally on agar plates. Each value represents the statistical mean of 15–20 seedlings. The surface of cotyledons were determined on digitalized pictures (30 seedlings per measurement) using NIH image software as described [50].

### Measure of Anthocyanin content

The anthocyanin content (30 seedlings per extraction) was measured using the method described in [35]. Seedlings were grown 4 days in continuous blue light (25 µmol m^−2^ s^−1^) on MS medium containing 1.5% sucrose. Data have been expressed as the difference of absorbance A_530 nm_–A_667 nm_ per 30 seedlings.

### Mapping

To precisely map the position of the *psi2* locus between the LFY3 and nga129 markers [26], polymorphism analysis of marker g2368 in the C24 and Ler ecotypes was used, following the method described in [51].

### cDNA clone of AtPP7

The complete coding sequence of AtPP7 was reconstructed by ligating the truncated cDNA present in the EST clone 122A12T7 (amplified using the primer F: TGCCTGTCAACGTCTTTG and the *Pst*I-site containing primer R: AATGTCATTGATTCTGCAGAT), with the rest of the coding sequence amplified from the genomic DNA by primers F: TTTAACGGATCCAATGGAAACTG and R: CAAAGACGTTGACAGGCA, using *Hinc*II digestion to create a merging restriction site. The construct was then cloned into pCR2.1 and amplified in *E. Coli*.

### Yeast 2-hybrid screen

To find peptides interacting with the AtPP7 protein, we used the method of interaction trap/two-hybrid system as described [52]. Two fragments of the AtPP7 coding sequence corresponding to the amino- and carboxy- termini were separately cloned in frame as bait fusions with the LexA protein in the yeast vector pEG202 as follows. The N-terminus fragment (233 bp) was first amplified by PCR from genomic DNA using the primers F: TTTACGGATCCAATGGAAACTG and R: GTCAAGATCATCGATATGAACG and cloned into pCR2.1, before being transferred as a *Bam*HI/*Not*I fragment in frame in the corresponding pEG202 insertion site. The C-terminus fragment (130 bp) was amplified from the EST clone 122A12T7 using a F primer containing a *Xma*I restriction site: GGGCCCTGCAGGCGCCTGATTCTCGGATCCTCAATTTCATAG and the R primer: ATCTGCAGAATCAATGACATT, and cloned as a *Xma*I/*Not*I fragment in pEG202. Both constructs were amplified in *E. coli* and sequenced for confirmation of correct frame insertion (using PCR primer CGTCAGCAGAGCTTCACCATTG). Either one of the resulting recombinant pEG202 bait-vectors, the *lacZ*-reporter gene-containing plasmid pSH 18–34, and the cDNA library of *Arabidopsis* present as GAL1-promoter fusions in pJG4-5 plasmid (a gift from Dr. H. Zhang, Texas Tech University) were introduced sequentially in yeast (strain EGY48) using the method of lithium acetate/polyethylene glycol transformation [53], followed by selection on sugar- and amino acid-depleted media. Positive colonies were grown in restrictive medium and submitted to quantitative measurement of the *lacZ* reporter gene activity using spectrophotometric measurement at 420 nm of the hydrolysis of ortho-nitrophenyl-ß-galactopyranoside (ONPG) as described [54].

### Production of transgenic Arabidopsis expressing AtPP7-GFP fusion

cDNA sequence of AtPP7 from our pCR2.5 clone was introduced into a binary vector (pBin19) containing a 35S promoter-GFP-nos coding sequence. The proper in-frame insertion between promoter and GFP sequence was controlled by sequencing. We used the floral dipping method [28] to transform *Arabidopsis psi2-DS1* mutants with *Agrobacterium* as described above. Transformants and homozygous lines were selected on Kanamycin, and tested for complementation in red light (normal hypocotyl elongation, no leaf HR). The localization of GFP fusion proteins was performed using a Leica DM 6000 B fluorescence microscope with a Leica CTR 6000 light source. For DAPI staining, the seedlings were observed in a ½ MS solution containing 0.5 µg/ml DAPI, 0.1% Triton X-100, and 20% glycerol.

### Protein interaction assay

AtPP7-GFP and control ankyrin-GFP lines expressing GFP fluorescent fusion in the nucleus were grown in darkness for 4 days and exposed to a 5 min red light pulse (10 µmol m^−2^ s^−1^) at 22°C. Tissues were ground in liquid N2 and proteins extracted in darkness under green light using Hepes buffer (50 mM, pH 7.9) containing 300 mM sucrose, 150 mM NaCl, 10 mM K-acetate, 5 mM EDTA, 0.5% Triton X-100 and proteinase inhibitor AEBSF (1 mM). Monoclonal anti-GFP antibodies coupled onto agarose beads (D153-8, MBL LTD, Nagoya, Japan) were used as prescribed by the manufacturer to immunoprecipitate AtPP7 and ankyrin fused to GFP. Protein extracts were incubated in darkness at 4°C during 5 hours on agarose beads blocked with 1 mg/ml BSA solution. Beads were then washed 5 times with fresh extraction buffer and resuspended in 2× FSB (Final Sample Buffer). Protein separation was performed by 12% SDS-PAGE. The gels were subsequently blotted on nitrocellulose membrane as described in [55] and blocked overnight in pH 7.2 saline phosphate buffer (PBS) containing 5% non-fat milk powder. We used monoclonal antibodies against GFP (BD Living Colors JL-8, Clontech catalog no. 8371-1) diluted 1/4,000 in PBS, 0.1% Tween 20, and 5% non-fat milk; anti-DET3 antibodies and anti-phyA antibodies were used as described in [55]. Anti-NDPK2 antibodies (gift from Dr Lucien Bovet) were used in 1/5000 dilution in PBS-Tween-milk buffer. Anti-mouse IgG and anti-rabbit IgG were used as secondary antibodies coupled to horse-radish peroxidase (Promega, Madison, USA) at a 1/10,000 dilution with 4 h incubation at room temperature. Secondary antibodies were detected by chemiluminescence using ECL reaction (Amersham).

### Production of double mutant *psi2-DS1 ndpk2* and triple mutant *phyA-phyB-psi2-DS1*

A plant containing the Ds transposon inserted in the *AtPP7* gene (*psi2-DS1* line, No-0 ecotype) was crossed with the *ndpk2* mutant insertion line of Ws-2 ecotype (gift from Prof. P.-S. Song). F2 seedlings were selected based on resistance to hygromycin and kanamycin and their progeny tested for homozygous double resistance; F3 progeny homogeneously growing on medium containing the two antibiotics were then selected and further characterized. Triple mutants *phyA-phyB-psi2-DS1* were obtained by crossing as described in [22].

## Supporting Information

Table S1 Complementation of the phenotypic hypersensitivity of the cab2::luciferase reporter gene induction by an intact genomic AtPP7.
(0.06 MB PDF)

Figure S1 Complementation of psi2 mutant by WT genomic AtPP7. Adult plants.
(0.52 MB PDF)

Figure S2 Nuclear localization of AtPP7-GFP and ankyrin-GFP in transgenic lines.
(1.57 MB PDF)

Figure S3 Complementation of the cotyledon enlargement phenotype of psi2 mutant by genomic AtPP7.
(0.05 MB PDF)

Figure S4 The complete AtPP7 protein (FLAG-tagged) interacts with a complete NDPK2 (6xHis-tagged) protein in vitro.
(0.08 MB PDF)

Figure S5 Anthocyanin content in blue light treated seedlings expressing various level of AtPP7.
(0.05 MB PDF)

Text S1 Supporting materials and methods.
(0.07 MB PDF)
