# Supplementary material for: The Protein Phosphatase 7 Regulates Phytochrome Signaling in Arabidopsis
Source: PLoS One. 2008 Jul 16;3(7):e2699. doi: 10.1371/journal.pone.0002699 (PMC2444027; doi:10.1371/journal.pone.0002699)
Supplement: Table S1 — Complementation of the phenotypic hypersensitivity of the cab2::luciferase reporter gene induction by an intact genomic AtPP7. (0.06 MB PDF) [file pone.0002699.s001.pdf]

Supporting Table S1. Complementation of the phenotypic hypersensitivity of the *cab2:Ω:luciferase* reporter gene induction by a WT genomic sequence of *AtPP7*. Luciferase activity in dark-grown seedlings was monitored 20 h after a pulse of light by luminescence video imaging.

Pulse and Fluence (μmol/m^{-2})	WT (*cab2:Ω:luc*)	*psi2-1* (*cab2:Ω:luc*)	*psi2-1* (*cab2:Ω:luc*) +genomic *AtPP7*
Dark	7.3[a] (3.9)[b]	9.8 (4.6)	8.5 (5.5)
Red light: 0.2	30.3 (5.6)	157.6 (12.4)	28.5 (4.8)
Red light: 1.0	43.1 (4.7)	225.9 (21.2)	47.3 (5.9)
Far-red light: 1.0	15.4 (3.2)	30.4 (5.5)	17.8 (3.4)
Far-red light: 13	21.3 (4.1)	72.5 (6.4)	20.4 (3.9)

([a])Counts per seedling/ 15 min. ([b])Standard deviation.
