# Supplementary material for: The Protein Phosphatase 7 Regulates Phytochrome Signaling in Arabidopsis
Source: PLoS One. 2008 Jul 16;3(7):e2699. doi: 10.1371/journal.pone.0002699 (PMC2444027; doi:10.1371/journal.pone.0002699)
Supplement: Figure S1 — Complementation of psi2 mutant by WT genomic AtPP7. Adult plants. (0.52 MB PDF) [file pone.0002699.s002.pdf]

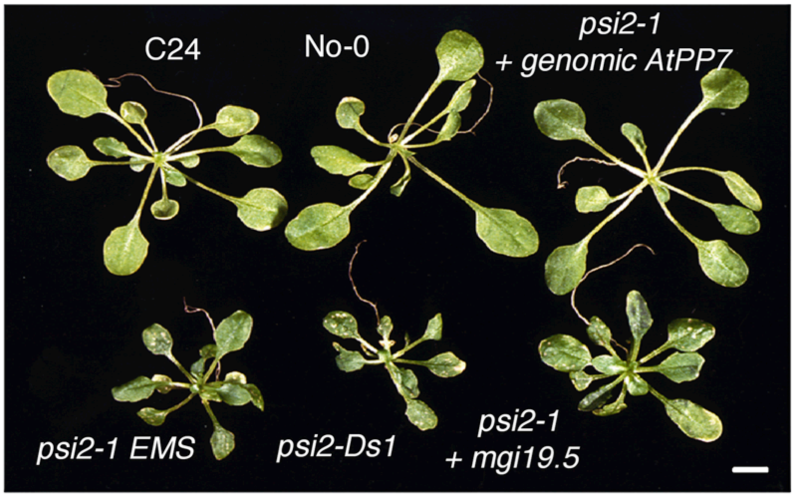

Supporting Figure S1

Morphology of WT plants, *psi2* mutants, transformed *psi2* mutant plant containing a WT genomic sequence of *AtPP7*, and a *psi2* mutant transformed with the control gene *MGI19.5*. Bar: 5 mm.
