# Supplementary material for: The Protein Phosphatase 7 Regulates Phytochrome Signaling in Arabidopsis
Source: PLoS One. 2008 Jul 16;3(7):e2699. doi: 10.1371/journal.pone.0002699 (PMC2444027; doi:10.1371/journal.pone.0002699)
Supplement: Figure S2 — Nuclear localization of AtPP7-GFP and ankyrin-GFP in transgenic lines. (1.57 MB PDF) [file pone.0002699.s003.pdf]

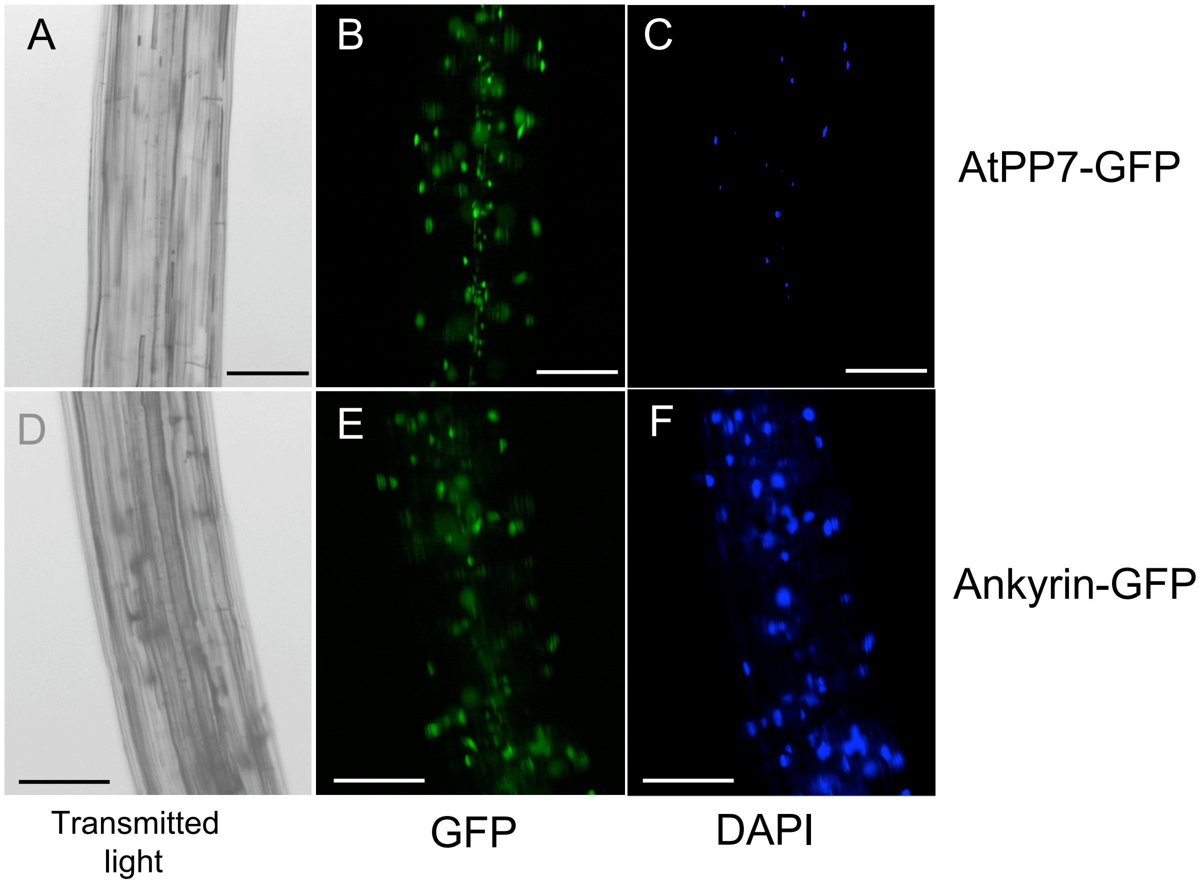

Supporting Figure S2

Localization of AtPP7-GFP fusion protein expressed in hypocotyl of transformed *psi2-DS*1 mutant (A, B, C) and of nuclear ankyrin-GFP fusion protein in control plants (D, E, F) by fluorescence microscopy (B, C, E, F). Seedlings were grown 4 days in darkness and prepared for microscopy under low green light. (C-F) DAPI staining show the position of nuclei. Bar: 200 µm.
