# Supplementary material for: The Protein Phosphatase 7 Regulates Phytochrome Signaling in Arabidopsis
Source: PLoS One. 2008 Jul 16;3(7):e2699. doi: 10.1371/journal.pone.0002699 (PMC2444027; doi:10.1371/journal.pone.0002699)
Supplement: Figure S3 — Complementation of the cotyledon enlargement phenotype of psi2 mutant by genomic AtPP7. (0.05 MB PDF) [file pone.0002699.s004.pdf]

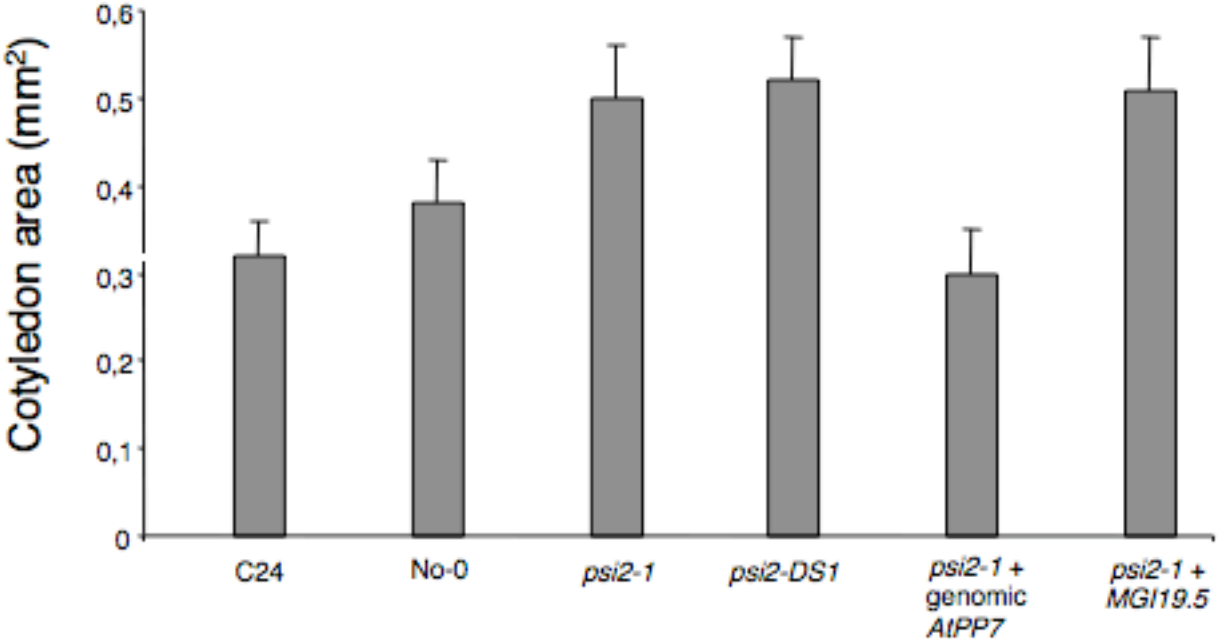

Supporting Figure S3

Cotyledon enlargement phenotype of *psi2* mutant is complemented with genomic *AtPP7*. Cotyledon surface of dark-grown seedlings were irradiated with 3 µmol m^{-2} s^{-1} continuous red light during 48 h.
