# Supplementary material for: The Protein Phosphatase 7 Regulates Phytochrome Signaling in Arabidopsis
Source: PLoS One. 2008 Jul 16;3(7):e2699. doi: 10.1371/journal.pone.0002699 (PMC2444027; doi:10.1371/journal.pone.0002699)
Supplement: Figure S4 — The complete AtPP7 protein (FLAG-tagged) interacts with a complete NDPK2 (6xHis-tagged) protein in vitro. (0.08 MB PDF) [file pone.0002699.s005.pdf]

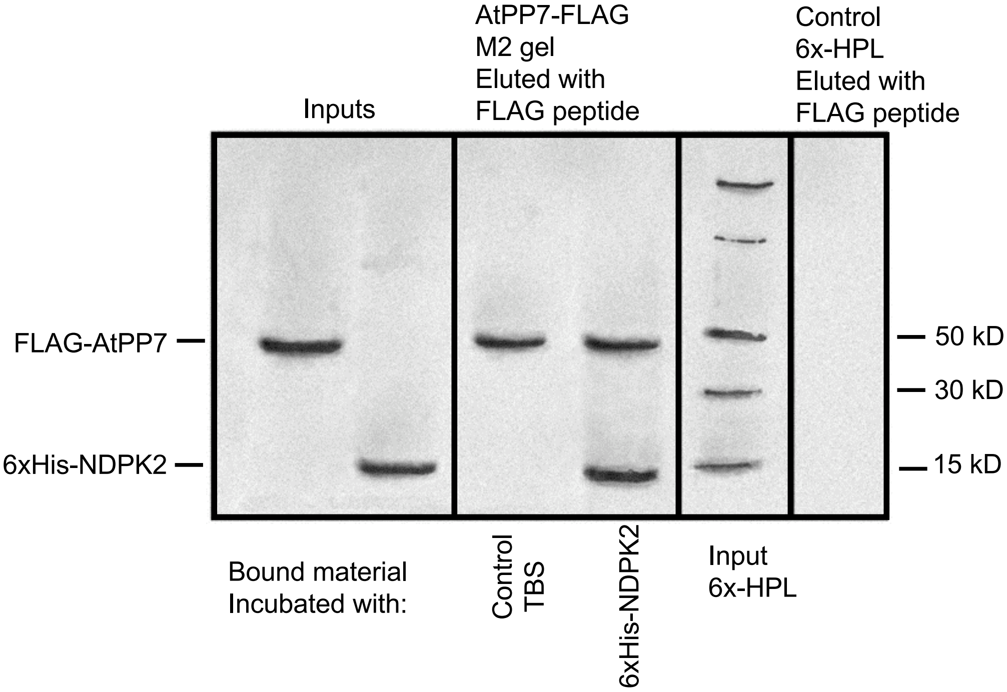

Supporting Figure S4

The complete AtPP7 protein (FLAG-tagged) interacts with a complete NDPK2 (6xHis-tagged) protein *in vitro*.
Recombinant proteins production and *in vitro* binding assays were conducted as described in Supporting Text S1: Material and Methods.
