# Supplementary material for: The Protein Phosphatase 7 Regulates Phytochrome Signaling in Arabidopsis
Source: PLoS One. 2008 Jul 16;3(7):e2699. doi: 10.1371/journal.pone.0002699 (PMC2444027; doi:10.1371/journal.pone.0002699)
Supplement: Figure S5 — Anthocyanin content in blue light treated seedlings expressing various level of AtPP7. (0.05 MB PDF) [file pone.0002699.s006.pdf]

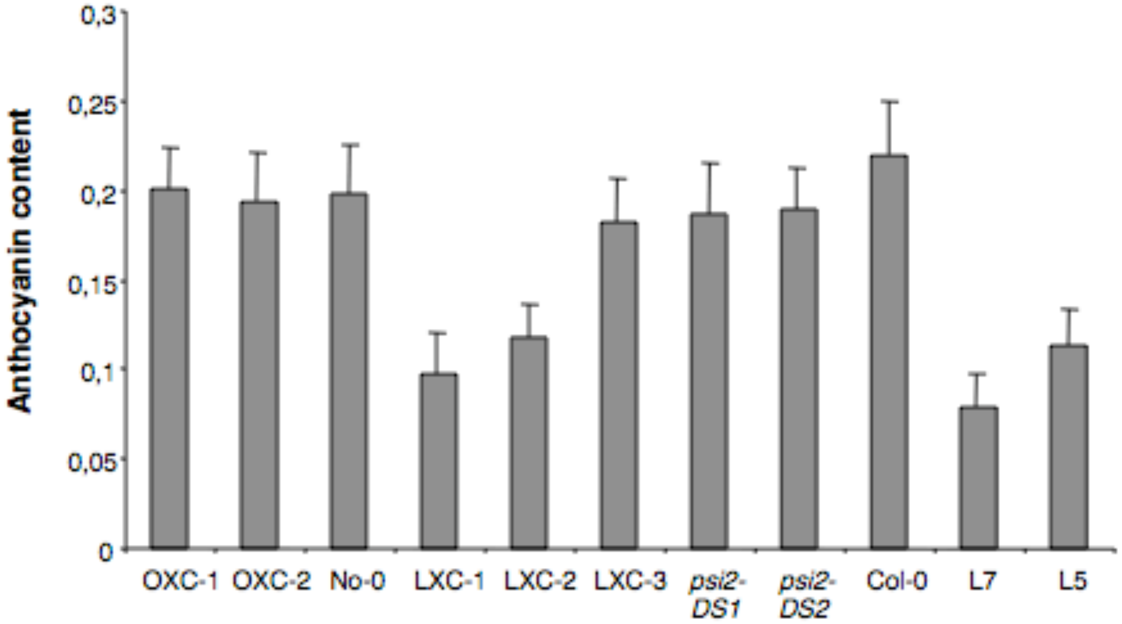

Supporting Figure S5

Anthocyanin content in blue light treated seedlings expressing various level of *AtPP7*. Seedlings of the genotype as before described were grown in continous blue light (25 µmol m^{-2} s^{-1}) for 4 days.
