# Supplementary material for: The Protein Phosphatase 7 Regulates Phytochrome Signaling in Arabidopsis
Source: PLoS One. 2008 Jul 16;3(7):e2699. doi: 10.1371/journal.pone.0002699 (PMC2444027; doi:10.1371/journal.pone.0002699)
Supplement: Text S1 — Supporting materials and methods. (0.07 MB PDF) [file pone.0002699.s007.pdf]

Supporting Text S1: Material and Methods

Production of recombinant NDPK2 and AtPP7 proteins. Recombinant NDPK2 protein was produced as a 6xHis-tag fusion protein using Qiagen Express Kit (containing three cloning vectors pQE30, pQE31 and pQE32 with alternative open reading frames). Using the restriction enzymes *Sal*I and *Hind*III, the *NDPK2* gene was excised from an EST plasmid clone (226E19T7) and inserted into the three different cloning vectors. The host cell strain was *E. coli* M15 (pREP4) from Qiagen Express Kit. Screening for NDPK2-6xHis fusion protein production using SDS-PAGE analysis, indicated pQE32 to be the right cloning vector. Synthesis of NDPK2-6xHis fusion protein in transformed *E. coli* liquid cultures was induced after $OD_{600} = 0.6$ with IPTG (Cf = 1 mM), and expression levels were monitored after 4.5 hr. Protein was isolated and purified from a 1-L bacterial culture. Batch purification with Ni-NTA resin-protein complex (support Sepharose® CL-6B, Qiagen Express Kit) of the polyHis NDPK2 fusion protein was made from pQE32 expression vector under native conditions.

Recombinant AtPP7-FLAG fusion protein was produced by using the FLAG expression system (Sigma). The coding sequence of *AtPP7* was excised from our pCR 2.1 recombinant plasmid using *EcoR*I and inserted into two cloning vectors, FlagShift 12 (periplasmic expression) and FlagShift 12C (cytoplasmic expression) containing a shift sequence. The *E. coli* host cell strain was INVα' (Invitrogen Cloning Kit).

Synthesis of AtPP7-FLAG protein was induced in liquid cultures after $DO_{600} = 0.4$ with IPTG (Cf = 1 mM) and the level of the expression was monitored after 3 hr. AtPP7-

FLAG was isolated from FlagShift 12 vector-containing bacteria by osmotic shock. Purification of the FLAG tagged AtPP7 protein produced was carried out using either ANTI-FLAG Affinity Gel M1 or ANTI-FLAG Affinity Gel M2 chromatography columns. Elution was conducted using EDTA. FLAG-tagged AtPP7 was purified from bacteria transformed with the recombinant Shift 12C Expression Vector using ANTI-FLAG Affinity Gel M2 chromatography, while elution was conducted with Glycine-HCl.

Protein electrophoresis and immunoblotting. SDS-polyacrylamide gel electrophoresis (SDS-PAGE) were performed at 20 mA using 2 x 1 mm separation gel (13% acrylamide) with a 5% acrylamide stacking gel. Proteins were electroblotted onto nitrocellulose membrane (Millipore) using usual transfer buffer at 4°C and 400 mA. 3 μl of 6X-HPL have been used as a positive anti-His staining control. For immunodetection of the NDPK2 protein and 6X-HPL (Qiagen Express Kit), the membrane was blocked with 3% BSA, while for AtPP7 protein immunodetection (Sigma Expression Kit), the membrane was blocked using 5% skim milk powder; in co-detection assays, 3% BSA was used. NDPK2 protein was detected using 0.1 μg/mL RGS and anti-Penta His Antibody (Anti His Selector Kit, Qiagen), and AtPP7 protein was detected using 10 μg/mL ANTI-FLAG Monoclonal Antibody M1 or M2. The secondary antibody was an alkaline phosphatase-conjugated goat antimouse immunoglobulin (DAKO, A/S, Denmark) used following the recommendation of the manufacturer.

***In vitro* test of protein-protein interaction**. ANTI-FLAG Affinity Gel M2 (800 μl) packed in a plastic column was saturated with recombinant FLAG-tagged AtPP7 protein

produced in bacterial periplasma (from 12C vector-containing cells). The column was washed with 36 ml TBS, and the ANTI-FLAG Affinity Gel M2 was then removed from the column, gently mixed with a pipette tip, divided into aliquots of 200 μl each, and repacked into 4 new columns. Two of these columns were then washed 5 times at room temperature with 3 ml TBS buffer containing 50 μg of His-tagged NDPK2 (purified as described), while the other two columns were washed with the same volume of control buffer and of a negative control buffer containing 7.5 μl 6xHis protein ladder (Qiagen). Unbound His-tagged NDPK2 was subsequently washed with 10 ml TBS, and the material bound to the gel was eluted 5 times with a single volume of 1 ml TBS or with a solution of 1 ml TBS containing dissolved FLAG peptide (100 μg/ml). The resulting eluates were concentrated 5 times and an aliquot of 50 μl of each sample was used for protein detection by immunoblotting as described above.
